# Detection of *Helicobacter pylori* and its virulence genes (*cag*A*, dup*A*,* and *vac*A) among patients with gastroduodenal diseases in Chris Hani Baragwanath Academic Hospital, South Africa

**DOI:** 10.1186/s12876-019-0986-0

**Published:** 2019-05-14

**Authors:** Ayodeji Idowu, Asisipho Mzukwa, Ute Harrison, Pia Palamides, Rainer Haas, Melvin Mbao, Razinah Mamdoo, Jonathan Bolon, Tolulope Jolaiya, Stella Smith, Reidwaan Ally, Anna Clarke, Henry Njom

**Affiliations:** 1 Department of Biochemistry and Microbiology, University of Fort Hare, Alice, Eastern Cape 5700 South Africa; 2 Chair of Medical Microbiology and Hospital Epidemiology, Max von Pettenkofer-Institute, Faculty of Medicine, LMU Munich, Munich, Germany; 3 Division of Gastroenterology, Chris Hani Baragwanath Academic Hospital (CHBAH), Soweto, Johannesburg, 2013 South Africa; 4 Department of Microbiology, University of Lagos, Akoka, Yaba Lagos Nigeria; 5 Molecular Biology Department, Nigerian Institute of Medical Research, Yaba, Lagos, Nigeria

**Keywords:** *H. pylori*, Peptic ulcer, PCR assay, Virulence genes, *cag*A, *dup*A, And *vac*A, Patients

## Abstract

**Background:**

The global prevalence of *H. pylori* approaches 50%, with prevalence rates between 20 and 40% in developed countries and up to 90% in Africa and other developing nations of the world. Development of *H. pylori*-associated diseases is determined by a number of virulence factors. This study aimed at determining the prevalence of *H. pylori* infections and virulence genes (*cag*A*, dup*A*, and vac*A); the relationship between virulence factors and gastroduodenal diseases among patients.

**Methods:**

Gastric biopsies were obtained from patients and cultured, DNA was extracted from cultured isolates and biopsies for PCR assay after which samples were investigated using standard laboratory procedures. Data of associated risk factors were obtained with the aid of questionnaires.

**Results:**

Of the 444 participants, *H. pylori* was detected in 115 (25.9%) from culture analysis and 217 (48.9%) by direct PCR method. Ninety-eight (85.2%) of the culture-positive patients were also detected by PCR giving an overall prevalence of 52.7% (234/444). The highest number of *H. pylori* isolates 76.9% (180/234) was obtained from patients suffering from pangastritis. The *Cag*A virulence gene was found in 62% (145/234), *dup*A in 53.4% (125/234) and *vac*A in 90.6% (212/234). *Vac*A genotype s1 m1 was the most prevalent [56.4% (132)] followed by s2 m2 [11.5% (27)], s2 m1 [10.3% (24)] and [s1 m2 9.4% (22)]. There was a significant association observed in *vac*A s1 and peptic ulcer disease, as well as *vac*A s1/m2 and gastric erosion (*P* < 0.05).

**Conclusion:**

The study revealed a significant association between virulence genes and the development of certain forms of gastric infections while the variations in *H. pylori* detection and the associated risk factors investigated in the study were not significantly related.

**Electronic supplementary material:**

The online version of this article (10.1186/s12876-019-0986-0) contains supplementary material, which is available to authorized users.

## Background

The global prevalence of *H. pylori* approaches 50% (approximately 4.4 billion individuals infected), with prevalence rates between 20 and 40% in developed countries and up to 90% in Africa and other developing nations of the world [1]. The prevalence of infection differs among the population of people and within countries in relation to race, ethnicity, and geographical location [2]. In South Africa, the high prevalence of infection with the pathogen is common in children and adults [3]. A prevalence of 87% was reported in the Eastern Cape among asymptomatic individuals [4]. Furthermore, the prevalence of 13.5 to 84.2% was reported in pediatric subjects of Bloemfontein, 66% in black children of KwaZulu/Natal, and 50.6% in Thohoyandou [5]. Variations in the prevalence rates have been attributed to factors such as low socio-economic conditions, poor hygiene, and overcrowding in residences [4]. Though mode of transmission is still unclear, many authors have suggested fecal-oral routes via contaminated water, food and unwashed hands [6, 7]. The smoking of cigarette, alcohol consumption, diet, occupational exposure, individual genetic trait have been demonstrated as risk factors associated with infection of *H. pylori* [8]. However, oftentimes the results are inconsistent, with some studies indicating no difference in prevalence compared to a control group [9], while others display a lower or a higher prevalence [10, 11].

Several diagnostic methods (both invasive and non-invasive) have been described to detect *H. pylori* infection for epidemiological studies [12]. In the current study, we used a culture method and polymerase chain reaction (PCR) assay to isolate and detect or confirm *H. pylori* respectively. The two diagnostic methods have been reported to be specific and sensitive while on several occasions regarded as gold standards [13]. However, due to the long duration of incubation and false negative results, the culture method is usually avoided. Some studies have embraced a seroprevalence approach, which detects the antibodies but is unable to differentiate passive and active infection.

On the basis of disease progression, development of ailment depends on bacteria strain, host body, and environmental factors. Induction and progression of *H. pylori*-associated diseases are determined by a number of virulence factors [14]. Among them, the cytotoxin-associated gene A (*cag*A), vacuolating cytotoxin gene A (*vac*A) and duodenal ulcer promoting gene A (*dup*A) virulence markers have been widely studied [15]. The *cag*A has been described as the first bacterial oncoprotein and probably the virulence factor with the most important potential of *H. pylori* [16] while *vac*A toxin plays a significant role in immune modulation as well as in the induction of gastric cancer [17]. Selection of virulence markers is vital when using them to determine the risk of diseases. For instance, the *cag*A, *dup*A and *vac*A genes have been considered important virulence factors in relation to gastroduodenal diseases both in children and adults in Brazil [18]. Furthermore, *cag*A and *vac*A genotypes have been implicated with gastric diseases in Pakistan [24]. Similarly, development of a duodenal ulcer in a South East Indian Population was seen as a result of the presence of *dup*A in *H. pylori* [20]. Elsewhere in Iran, infection with *H. pylori dup*A negative strains was associated with high risk of development of stomach ulcer and cancer [21].

In Soweto, South Africa, existing data on the subject are few [22]. Thus, there is a need for a recent study to provide information on *H. pylori* virulence factors and their roles in the development of gastroduodenal diseases. Therefore, this study was aimed at determining the prevalence of *H. pylori*, detect the presence of *cag*A, *vac*A and *dup*A virulence genes in patients at Chris Hani Baragwanath Academic Hospital and to analyze the relationship between these virulence genes and gastroduodenal disease development. The study also investigated the risk factors associated with gastroduodenal diseases among participants.

## Methods

### Patient recruitment

Patients were recruited in the gastrointestinal tract (GIT) unit of the Chris Hani Baragwanath Academic Hospital (CHBAH) between August 2017 and February 2018. Only those who gave consent were enrolled. The study included those with stomach affliction and referred for a non-sedated upper gastrointestinal endoscopy excluding patients recently on antibiotics and other eradication therapies in the last 3 months. Research questionnaires [Additional file 1] were administered to volunteered participants. The hospital (CHBAH) is a public health institution located in the Soweto area of Johannesburg (GPS coordinates 26.2612^o^S, 27.9426°E). It is known as the third largest hospital in the world with approximately 3200 beds. The hospital has a well functional GIT clinic which enrolls the highest number of patients in the country (over 2000 procedures annually). It is run by the Gauteng Provincial Health Authority and also a Teaching Hospital affiliated to the Medical School of The University of the Witwatersrand.

### Collection of biopsies

A consultant gastroenterologist performed the endoscopy. Patients with different gastroduodenal related pathologies were sampled. Gastric biopsies were obtained from antrum and corpus of the stomach using jumbo forceps (Boston Scientific South Africa (Pty) Ltd.) and transferred into vials containing portagerm pylori [23] and cultured within 4 h after collection.

### Isolation of *H. pylori*

Biopsy specimens were aseptically rolled over the surface of Columbia blood agar base plates under a biological safety cabinet (Thermo Scientific). The agar (Oxoid CM0331) was supplemented with 7% horse serum (Oxoid SR0048), 1% vitamin mix (Isovitalex), and an *H. pylori* selective supplement (Dent, SR0147E Oxoid) comprising of amphotericin B (2.5 mg), trimethoprim (2.5 mg), vancomycin (5.0 mg), and cefsulodin (2.5 mg). The plates were incubated at 37 °C in an atmosphere of 85% N_2_, 10% CO_2_ and 5% O_2_ for 4–10 days. Presumptive *H. pylori* colonies were identified as small, round, translucent, Gram-negative and positive for catalase, oxidase and urease tests. The confirmed isolates were Frozen in brain heart infusion broth (BHI) containing 20% glycerol and stored at − 80 °C for future use.

### DNA extraction

Total genomic DNA was extracted from biopsy tissues and cultured isolates using a commercial kit (QIAamp DNA Mini Kit; Qiagen, Hilden, Germany), according to the manufacturer’s instructions. Briefly, biopsies were lysed in 180 μl of ATL buffer and 20 μl of proteinase K at 56 °C for overnight incubation. Two hundred microliter of AL buffer was added to the lysate and samples were incubated for 10 min at 70 °C. After the addition of 200 μl absolute ethanol, lysates were purified over a QIAamp column as specified by the manufacturer. The column was washed stepwisely with 500 μl buffer AW1 and buffer AW2, after which an ultra-pure DNA product was eluted for PCR assay.

### Molecular confirmation of *H. pylori*

PCR was performed on extracted DNA from biopsies using primers specific for *H. pylori* 16S rRNA under the following conditions: Initial denaturation of 95 °C for 5 mins and 35 cycles of 95 °C for 30 s, 54 °C for 30 s and 72 °C for 30 s and a final extension time of 72 °C for 10 min. The PCR amplification was performed using a thermocycler system (Bio-Rad Thermal cycler, Singapore). Each 25 μl PCR reaction mixture contained 12.5 μl PCR master mix (Promega, GoTag® Green Master Mix, USA), 0.5 μl each of primer (Metabion, Planegg, Germany), 5 μl of template DNA and 6.5 μl of PCR grade water. For each PCR experiment, appropriate positive and negative controls were included. The *H. pylori* strain J99 and nuclease-free water were used as positive and negative controls respectively. To detect the amplified product, 5 μl of amplicons was visualized by electrophoresis through a 1.5% agarose gel (Merck, SA) at 100 V for 40 min in 1X TAE buffer and stained with ethidium bromide (500 ng/ml) (Sigma-Aldrich, USA) using the gel documentation system (Alliance 4.7, France). Identification of the bands was established by comparison of the band sizes with molecular weight markers of 100-bp (Thermo Scientific, (EU) Lithuania). Samples were considered positive when the visible band was the same size as that of the positive control DNA. The primer for 110 bp product of the 16SrRNA sequence represented by the forward primer sequence: 5′-CTGGAGAGACTAAGCCCTCC-3′ and the reverse one: 5′-ATTACTGACGCTGATTGTGC-3′ [24].

### Detection of virulence genes

Virulence genes *cag*A, *dup*A and *vac*A (subtypes: s1, s2, m1, and m2) were detected using PCR specific primers (Table 1) with the same amplification conditions and assay protocol as earlier described.

**Table 1:** Primer sequences for PCR detection of virulence genes

Target gene	Primer pair (5′-3′)	Amplicon length (bp)	Control strains	References
*cag*A	F: ACCGCTCGAGAACCCTAGTCGGTAATGGG R: *CAG*GTACCGCGGCCGCTTAAGATTTTTGGAAACCAC	981	J99	[25]
*vacA*s1/S2	F: ATGGAAATACAACAAACACAC R: CTGCTTGAATGCGCCAAAC	259/286	J99/Tx30a	
*vacA*m1	F: GGTCAAAATGCGGTCATGG R: CCATTGGTACCTGTAGAAAC	290	J99	
*vacA*m2	F: CATAACTAGCGCCTTGCAC R: GGAGCCC*CAG*GAAACATTG	352	B8	
*dupA*	F: GACGATTGAGCGATGGGAATAT R: CTGAGAAGCCTTATTATCTTGTTGG	971	P12/J99	[26]

### Statistical analysis

Data were analyzed by a two by two table statistics and chi-square test to determine the association between *H. pylori* positivity and epidemiological risk factors, as well as virulence factors and gastroduodenal disease diagnosis using the SPSS statistical software package version 18.0 (SPSS, Inc., Chicago, IL) and Open Source Epidemiology Statistics for Public Health Version 3.01. *P* values < 0.05 were considered statistically significant.

## Results

### Culture and PCR analysis

**Fig. 1:**
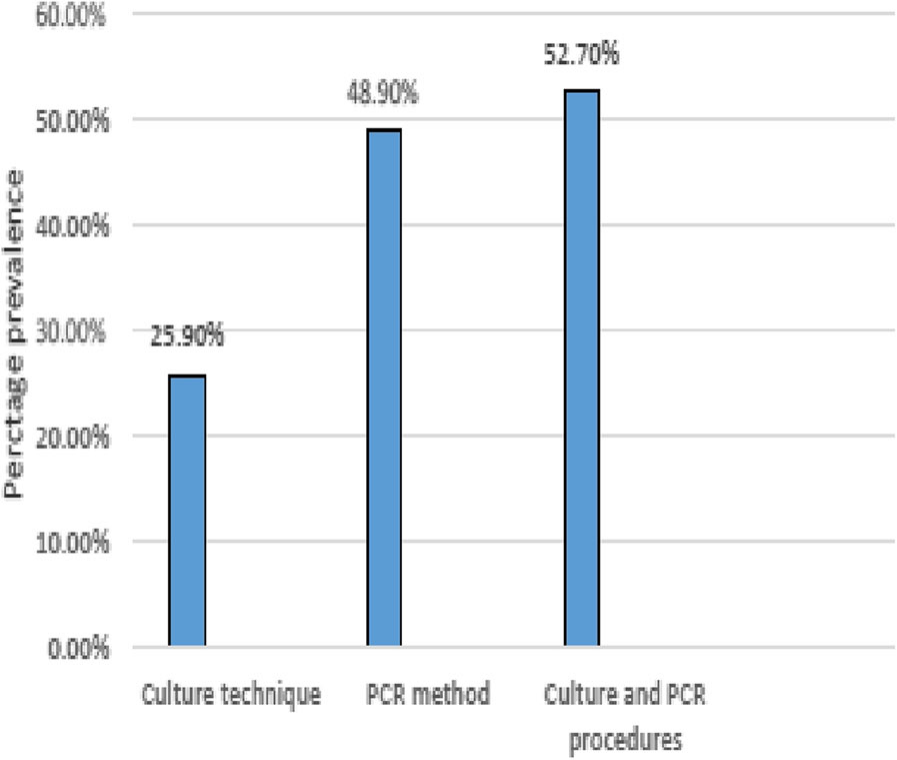
*H. pylori* prevalence by culture and PCR methods

**Fig. 2:**
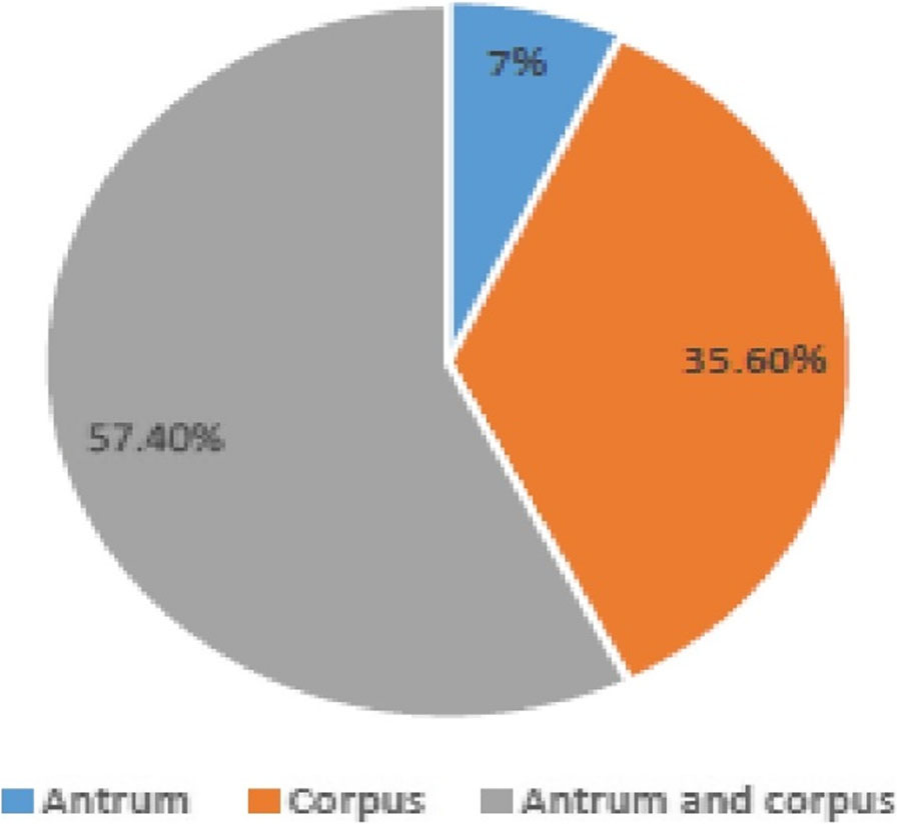
Positive culture result analysis from antrum and corpus specimens

**Fig. 3:**
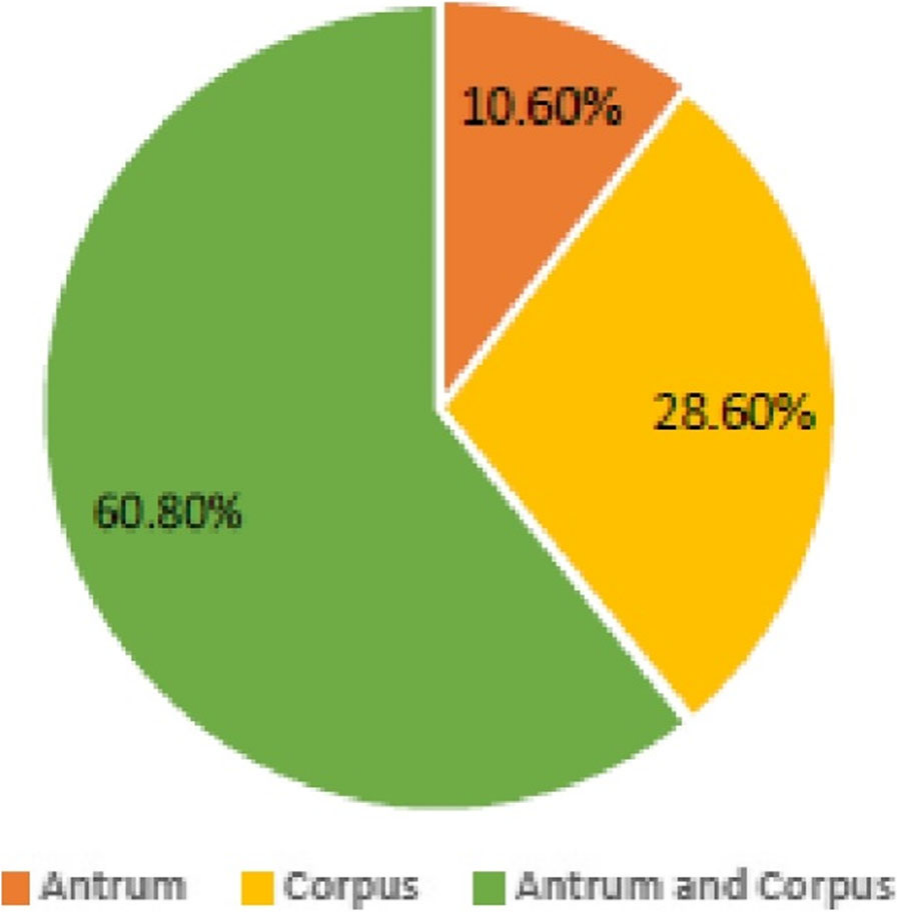
Positive PCR result analysis from antrum and corpus specimens

**Fig. 4:**
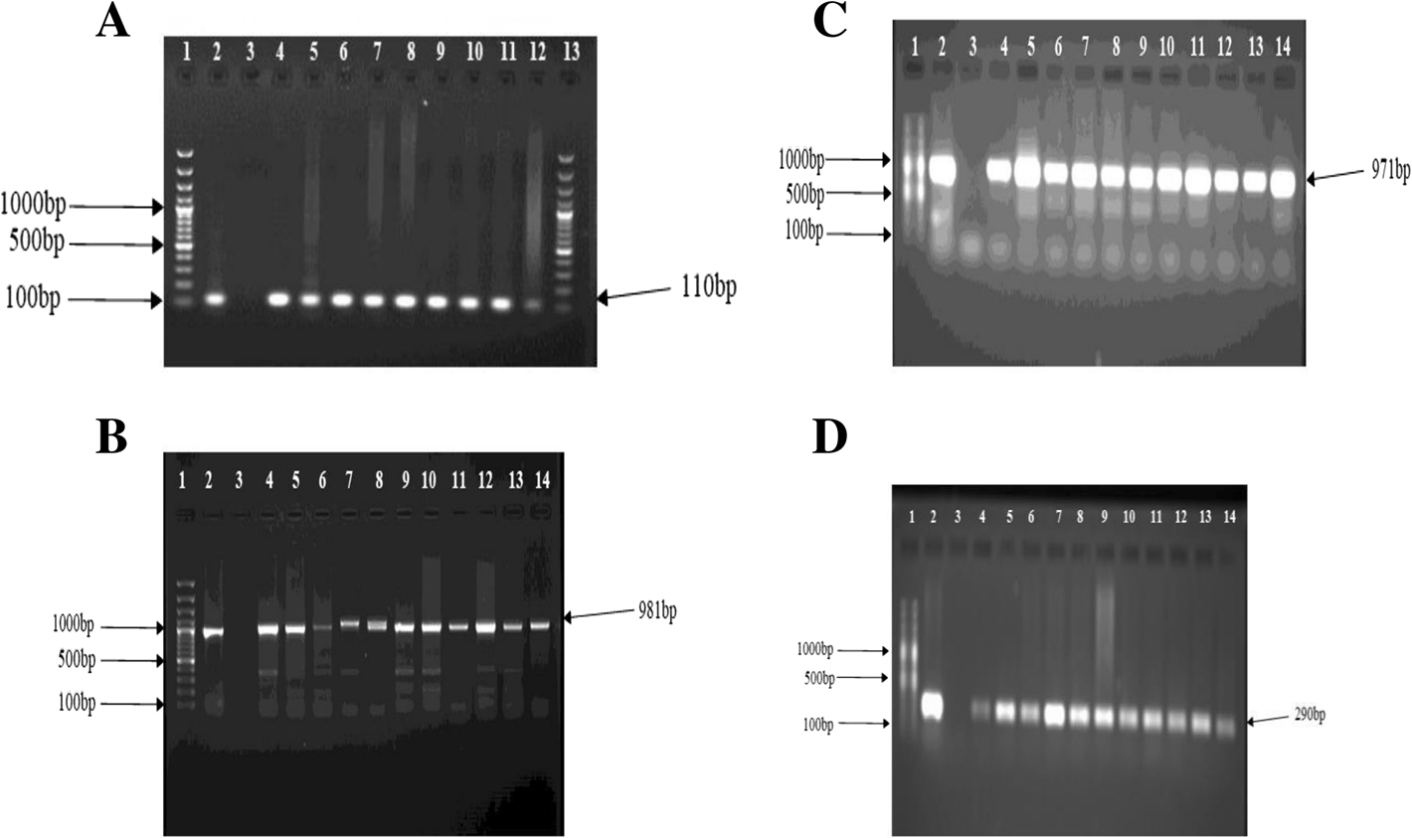
**a** Agarose (1.5%) gel electrophoresis of PCR products of the amplified 110 bp 16S rRNA gene. Lane 1 and 13: molecular weight markers (100 bp); lane 2: positive control (J99); lane 3: negative control (nuclease-free water); lanes 4–12: positive isolates. **b** Agarose (1.5%) gel electrophoresis of PCR products for *cag*A (981 bp) detection. Lane 1: 100 bp gene ruler, Lanes 2: *cag*A positive control, lane 3: negative control, lanes 4–14: *cag*A positive isolates. **c** Agarose (1.5%) gel electrophoresis of PCR products for *dup*A (971 bp) detection. Lane 1: 100 bp gene ruler, Lanes 2: *dup*A positive control, lane 3: negative control, lanes 4–14: *dup*A positive isolates. **d** Agarose (1.5%) gel electrophoresis of PCR products for *vac*A m1 (290bp) detection. Lane 1: 100 bp gene ruler, Lane 2: *vac*A m1. positive control, lane 3: negative control, lanes 4–14: *vac*A m1 positive isolates

Of the 444 recruited patients, *H. pylori* was obtained in 115 (25.9%) from culture analysis in either antrum or corpus or both biopsies. Two hundred and seventeen patients (48.9%) were positive by the PCR technique (from either antrum or corpus or both biopsies). Ninety-eight (85.2%) of the patients positive for culture were detected in PCR, giving an overall prevalence of 52.7% (i.e. 234/444) (Fig. 1). Of the 115 isolates, 8 (7%) were obtained from only antrum biopsies, 41 (35.6%) from corpus tissues and 66 (57.4%) from both specimens (Fig. 2). Overall, antrum had 16.7% (74/444) culture prevalence while corpus had 24.1% (107/444). Also, from the 217 *H. pylori* positive by the PCR assay, 132 (60.8%) were positive for both antrum and corpus, 23 (10.6%) for antrum biopsy only and 62 (28.6%) for corpus only (Fig. 3). Therefore, PCR percentage prevalence for antrum was 34.9% (155/444) and corpus was 43.7% (194/444). Some of the agarose gels showing PCR products are presented in Fig. 4a-d.

### Participant gender and *H. pylori* status

Of the 444 participants, 284 (64%) were females and 160 (36%) males. The age range was between 15 and 100 years, mean age was 52.7 ± 16.3 years, and 25% (109/444) of subjects were below 40 years. Of the 284 females, 150 (52.8%) were *H. pylori* positive while in males, 84 (52.5%) of 160 were positive.

### Influence of epidemiological and socioeconomic risk factors

Table 2 highlights epidemiological risk factors and socioeconomic conditions in *H. pylori* positivity. Patients with a family history of peptic ulcer and gastric cancer were 74 (16.7%) and 39 (16.7%) were positive for *H. pylori*. Two hundred and fifty-six (57.7%) patients were living with more than three people and *H. pylori* was isolated in 141 (60.3%) of this number. Thirteen (2.9%) patients were drinking from untreated water sources and 6 (2.6%) were *H. pylori* positive. Patients who smoke and drink alcohol either regularly or occasionally were 158 (35.6%) and 90 (38.5%) of them were positive for *H. pylori*. Twenty-four (5.4%) patients were involved in *H. pylori* risk occupations (e.g. people working directly with *H. pylori-*infected individuals such as medical practitioners) and 14 (6%) were tested positive for *H. pylori*. Patients without formal education were 32 (9.8%) and 18 (7.7%) were *H. pylori* positive. Fifty-two (11.7%) patients had traveled outside the country within the last 5 years for more than 1 month and 25 (10.7%) tested positive. However, highlighted possible risk factors and socioeconomic conditions were not statistically significant (*P* > 0.05) (Table 2).

**Table 2:** Epidemiological risk factors and socioeconomic conditions of *H. pylori* infection

Variables and categories	Total participants (%) *n* = 444	*H. pylori* positive (%) *n* = 234	*H. pylori* negative (%) *n* = 210	Odd ratio (95% CI)	X2 test	*P*-value
Family history of ulcer and cancer						
Yes	74 (16.7)	39 (16.7)	35 (16.7)	1	0	> 0.99
No	370 (83.3)	195 (83.3)	175 (83.3)	(0.61–1.65)		
Living with more than 3 people						
Yes	256 (57.7)	141 (60.3)	115 (54.8)	1.25	1.37	0.24
No	188 (42.3)	93 (39.7)	95 (45.2)	(0.86–1.83)		
Drinking from untreated water sources (e.g. stream, river)						
Yes	13 (2.9)	6 (2.6)	7 (3.3)	0.76	0.23	0.63
No	431 (97.1)	228 (97.4)	203 (96.7)	(0.25–2.31)		
Smoking and drinking of alcohol						
Yes	158 (35.6)	90 (38.5)	68 (32.4)	1.30	1.78	0.18
No	286 (64.4)	144 (61.5)	142 (67.6)	(0.88–1.93)		
Involved in *H. pylori* risk occupation						
Yes	24 (5.4)	14 (6)	10 (4.8)	1.27	0.32	0.57
No	420 (94.6)	220 (94)	200 (95.2)	(0.55–2.93)		
No Formal education						
Yes	32 (7.2)	18 (7.7)	14 (6.7)	1.17	0.17	0.68
No	412 (92.8)	216 (92.3)	196 (93.3)	(0.56–2.41)		
Travelled outside the country within last 5 years for more than 1 month						
Yes	52 (11.7)	25 (10.7)	27 (12.9)	0.81	0.51	0.48
No	392 (88.3)	209 (89.3)	183 (87.1)	(0.45–1.45)		

### Virulence gene detection and endoscopic conditions in *H. pylori* positive individuals

The endoscopy grading in 444 participants showed normal mucosa in 17 (3.8%) individuals, pan gastritis in 338 (76.1%), peptic ulcers in 55 (12.4%), gastric erosion in 23 (5.2%), gastric cancer in 3 (0.7%) and haemorrhages and polyps in 8 (1.8%). The highest number of *H. pylori* positives [180 (76.9%)] was obtained in pan gastritis patients, followed by participants with peptic ulcer 31 (13.2%). No *H. pylori* isolate was obtained in participants with gastric cancer. Virulence genes were determined for all confirmed *H. pylori* positive patients obtained from culture isolates and biopsy DNA out of which the *cag*A gene was detected in 62% (145/234), *dup*A in 53.4% (125/234), *vac*As1 in 66.2% (155/234), *vac*As2 in 20.1% (47/234), *vac*Am1 in 69.2% (162/234) and *vac*Am2 in 21.4% (50/234) strains.

The *cag*A virulence gene was highest [111 (76.5%)] in pan gastritis patients followed by individuals with peptic ulcer [17 (11.7%)], while participants with haemorrhages and polyps had the lowest *cag*A detection [2 (1.4%)]. In this study, virulent allelic combination s1/m1 was predominant and found in 132 (56.4%) of the *H. pylori* strains, the s1/m2 was detected in 22 (9.4%) and the other genotypes, s2/m1 and s2/m2 were recorded in 24 (10.3%) and 27 (11.5%) strains respectively. Virulence genes *cag*A, *dup*A, *vac*A s1/m1 were detected in all disease conditions except gastric cancer where *H. pylori* was not found. Detection of *vac*A s1 was significantly high 26 (16.8%) in patients with peptic ulcer disease (*P* = 0.036). Similarly, *vac*A s1/m2 was considerably high in patients with gastric erosion (*P* = 0.0029) (Table 3). Overall, 90.6% (212/234) of *H. pylori* strains were positive for *vac*A genotypes (data not exclusive but form unions and intersections).

**Table 3:** Detection and distribution of *H. pylori* and its virulence genes in patients with various gastroduodenal disease outcomes

	Disease conditions						
	Normal mucosal (%)	Pan gastritis (%)	Peptic ulcer (%)	Gastric erosion (%)	Hemorrhages and polyps (%)	Gastric cancer (%)	Total (%)
	*n* = 17 (3.8)	*n* = 338 (76.1)	*n* = 55 (12.4)	*n* = 23 (5.2)	*n* = 8 (1.8)	*n* = 3 (0.7)	*n* = 444 (100)
*H. pylori* positive	9 (3.8)	180 (76.9)	31 (13.2)	11 (4.7)	3 (1.3)	0 (0)	234 (100)
*cag*A	7 (4.8)	111 (76.5)	17 (11.7)	8 (5.5)	2 (1.4)	0 (0)	145 (100)
*dupA*	5 (4.0)	94 (75.2)	15 (12.0)	9 (7.2)	2 (1.6)	0 (0)	125 (100)
*vacA*s1	8 (5.2)	111 (71.6)	26 (16.8) *	7 (4.5)	3 (1.9)	0 (0)	155 (100)
*vacA*s2	1 (2.1)	38 (80.9)	5 (10.6)	3 (6.4)	0 (0)	0 (0)	47 (100)
*vacA*m1	6 (3.7)	123 (75.9)	23 (14.2)	7 (4.3)	3 (1.9)	0 (0)	162 (100)
*vacA*m2	3 (6.0)	37 (74.0)	7 (14.0)	3 (6.0)	0 (0)	0 (0)	50 (100)
*vacA*s1/m1	6 (4.5)	97 (73.5)	20 (15.2)	6 (4.5)	3 (2.3)	0 (0)	132 (100)
*vacA*s1/m2	2 (9.1)	13 (59.0)	2 (9.1)	5 (22.7) *	0 (0)	0 (0)	22 (100)
*vacA*s2/m1	0 (0)	19 (79.2)	3 (12.5)	2 (8.3)	0 (0)	0 (0)	24 (100)
*vacA*s2/m2	1 (3.7)	22 (81.5)	2 (7.4)	2 (7.4)	0 (0)	0 (0)	27 (100)

*Significant (*P* < 0.05)

### Association between *cag*A, *dup*A and *vac*A genotypes in *H. pylori* strains

Table 4 shows the association between *cag*A, *dup*A and *vac*A genotypes in *H. pylori* strains. The *vac*A s1 was found to be significantly high (*P* = 0.000) in the *cag*A and *dup*A positives patients. Similarly, *vac*A m1 positive individuals recorded a significantly high *cag*A 123 (75.9%) and *dup*A 100 (61.7%) genotypes. Furthermore, there was a high detection rate of *cag*A 82.6% (109/132) and *dup*A 63.6% (84/132) in the *vac*A s1/m1 positive patients. The rest of the *vac*A genotypes were not significantly high in *cag*A and *dup*A positive individuals.

**Table 4:** Simultaneous detection of *vac*A, *cag*A and *dup*A in *H. pylori* strains

S/N	*vac*A genotypes (%) *n* = 212	*cag*A positive (%) *n* = 145	*dup*A positive (%) *n* = 125
1	s1 (*n* = 155)	122 (78.7) *	97 (62.6) *
2	s2 (*n* = 47)	19 (40.4)	21 (44.7)
3	m1 (*n* = 162)	123 (75.9) *	100 (61.7) *
4	m2 (*n* = 50)	22 (44.0)	25 (50.0)
5	s1/m1 (*n* = 132)	109 (82.6) *	84 (63.6) *
6	s1/m2 (*n* = 22)	13 (59.1)	12 (54.5)
7	s2/m1 (*n* = 24)	12 (50.0)	13 (54.2)
8	s2/m2 (*n* = 27)	10 (37.0)	12 (44.4)

*significance (*P* < 0.05)

## Discussion

Various diagnostic methods of detecting *H. pylori* have been proposed. The choice usually depends on the availability of materials, sampling population, condition of patients, and competency or experience of the investigator. In this study, the two methods complement each other for diagnosis and analysis. The choice of culture technique was based on a high record of specificity and availability of isolates for antibiotic susceptibility test. Similarly, PCR assay was chosen due to its high sensitivity reported by many authors [13]. Comparing both results, we observed a higher prevalence rate (48.9%) in the PCR method than culture (25.9%), given a difference of 23%. The reason for the wide margin in the outcome of the two methods could be as a result of non-viability of some strains of *H. pylori* in agar medium as reported by some authors [27]. In this study, 85.2% of the culture-positive individuals were detected by the PCR assay. This outcome is below the expected 100% positivity. This outcome may be due to different biopsies used for the analysis and sampling error occurs when the pathogen is not evenly distributed within the mucosa.

The overall prevalence of 52.7% obtained in the current study is relatively low compared to some other previous studies in developing nations where higher prevalence figures were recorded [2, 28]. However, the outcome is not uncommon in South Africa as a lower rate 50.6% previously reported elsewhere in the country [5]. Furthermore, lower prevalence rates in some developing nations have been recently reported in Nigeria [25] Thailand [29], Indonesia [10] and Iran [30].

The bacterium is known to possess a wide degree of genomic and allelic diversity [31]. This special feature enables the organism to play an active role in the multiple gastric disorders in infected patients globally. One of the factors responsible for the multiple clinical presentations in individuals is a function of the bacterial virulence factors. This study, therefore, focused on the important virulence genes inducing gastroduodenal diseases among patients. The *cag*A and *vac*A have been related to inducing gastric adenocarcinoma, mucosal associated lymph tissue (MALT)-lymphoma, and peptic ulcer disease (PUD) in patients [32] while *dup*A is associated with duodenal ulcer [33]. Studies have revealed that the frequency and/or severity of gastroduodenal disease related to *H. pylori* varies geographically [34, 35]. This proposition is somewhat due to a difference in the distribution pattern of virulence markers in circulating strains of the organism. For instance, it has been reported that East-Asian-type *cag*A with repeated EPIYA segment sequence A-B-D has a higher binding affinity than the western-type *cag*A with sequence A-B-C resulting in higher risk of peptic ulcer and gastric cancer [36]. Furthermore, a low incidence of gastric cancer and peptic ulceration has been reported in a population of a high percentage of *vac*A m2, *dup*A negative and *cag*A negative *H. pylori* strains [37].

In this study, to establish a possible association between the presence of the major virulence factors and disease outcome among patients attending CHBAH, Soweto, we analyzed the status of *cag*A, *vac*A and *dup*A genotypes of 234 *H. pylori* strains. The majority of strains were *vac*A 90.6% (212/234), followed by *cag*A 62% (145/234) and *dup*A 53.4% (125/234). Among the *vac*A genotype combinations, *vac*A s1/m1 was the highest at 56.4% (132/234).

*Cag*A is one of the most studied virulence genes of *H. pylori* and this toxic protein has a molecular size of 120 to 145 kDa and is found on the *cag*-PAI. The strains that carry the PAI are known to be more virulent than those lacking it [38]. The prevalence of 62% *cag*A positive strains obtained in our study is similar to researches conducted in Tunisia and Morocco where 61.6 and 61.2% *cag*A were reported respectively [39, 40]. However, the outcome is lower compared to a higher prevalence *cag*A previously reported elsewhere in South Africa. For example, in Eastern Cape, South Africa, 90% *cag*A positive strains were reported [41], while in Gauteng, 87% cases of *cag*A positive were found among asymptomatic children age between 6 and 15 years in another study. Globally, in Taiwan, 83% *cag*A positive strains were found in isolates from patients with chronic gastritis and peptic ulcer [42]. Among Turkish patients with dyspepsia, 74% *cag*A was detected [43] and 85% *cag*A was reported among Alaskans (USA) [44]. Also, 93% in Nigeria [45] and 96% in Indian [19]. On the other hand, a lower prevalence of *cag*A has been documented elsewhere such as Cuba [46], Pakistan, Egypt, Israel and Jordan [19].

Vacuolating cytotoxin (*vac*A) has been implicated to play a major role in gastroduodenal disease progression. The gene is a pore-forming toxin secreted through an auto transporter. The mechanism of its toxigenic effect occurs by binding to the receptor of the eukaryotic cell lipid sphingomyelin. The gene targets the mitochondria where it induces apoptosis and formation of large intracellular *vac*uoles. VacA polymorphisms are divided into signal, intermediate, middle and deletion regions. Of all the allelic combinations, the *vac*A s1/m1 alleles are the most virulent, while the s1/m2, s2/m1 and s2/m2 genotypes demonstrate little to no pathogenicity. Our study showed *vac*A s1 to be predominant, which is similar to findings reported elsewhere such as Eastern Cape South Africa [41], Thailand [29] and Indian [20]. In the same vein, Findings in Alaska (USA) [44], Cuba [46] and Morocco [40] showed the dominance of *vac*As1 subtypes and its link with disease status. On the other hand, a lower prevalence of s1 type allele has been reported in Jordan [47] as well as Iran [48].

Generally, our finding shows a high prevalence of *vac*A genotypes (90.6%) predominantly of the subtypes s1 and m1 which makes patients likely to be more prone to *H. pylori*-associated diseases. The absence of *vac*A in 22 strains in the study could be a result of the genetic structure of the strains or exposure to adverse stomach conditions [49]. In the study, the subtype *vac*A m1 (69.2%) were more than *vac*A m2 (21.4%). The outcome is in line with findings of other authors who reported a higher prevalence of *vac*A m1 than m2 [50–52]. However, other studies have reported contrary results [41, 43]. Numerical data of *vac*A polymorphs differ from strains due to genetic composition and geographical location of the organism. For example, m1 genotype appears more than m2 in African population while the two subtypes are almost equally distributed within Europe and Latin America [53].

This result compared to a study conducted in Nigeria is lower in terms of the presence of *cag*A and *vac*A virulence genes among *H. pylori* strains [25]. However, no correlation with pathology could be observed in the Nigerian study contrary to our findings which showed a significant relationship between *vac*As1 and peptic ulcer as well as *vac*As1/m2 and gastric erosion. The reason probably could be the influence of host genetic make-up and environmental conditions [54]. In the same vein, our finding is parallel with a study conducted in Brazil which showed that *vac*A s1/m1 genotype may be considered an important virulence factor in the development of gastric diseases [18].

Duodenal ulcer promoting gene (*dup*A) has been considered as a marker for the peptic ulcers by some authors [18] but in our study, the association could not be linked. In line with our study, some authors were unable to regard *dup*A as a predictor of duodenal ulceration in Belgium, South Africa, China and North America [55]. We recorded 53.4% *dup*A positive isolates in our study, while the lower prevalence of *dup*A (18.8%) was reported in Northern Iraq [26] and as well as 39% in Iran [56].

*H. pylori* infection leads to several gastroduodenal diseases including gastric cancer. In this study, few cases of gastric cancer (3/444) occurred, but none was accompanied with *H. pylori* infection. Due to limited data of patients with gastric cancer, though not uncommon in Africa, we cannot extrapolate gastric cancer and *H. pylori* negativity. Some studies have described factors such as viral infection, habit, immunological disorders and gene mutations as the aetiology of gastric cancer [57]. This study contains findings from gastroenterology reports of patients, while detailed pathological examination by histological grading of stomach biopsies was omitted. We, therefore, hope to include histological examination in the future study to enable comparison with gastroenterology diagnosis.

## Conclusions

The current study showed a vital correlation between *vac*As1 and the development of peptic ulcer disease as well as *vac*As1/m2 and gastric erosion. In South Africa and other developing nations, the occurrence of *H. pylori* virulence factors is common, but their involvement as disease markers is not really pronounced. Studies of *H. pylori* virulence factors in South Africa could be important for a clinical and epidemiological survey to better understand the disease pathology.

## Additional files

Additional file 1: Patient questionnaire. Contains patients investigation form, gastroenterology result sheet and consent form. (PDF 420 kb)

Additional file 2: Ethics certificate. Contains ethics certificates from the CHBAH and University of Fort Hare. (PDF 154 kb)

## Abbreviations

BHI: Brain heart infusion
*Cag* PAI: *cag* Pathogenicity Island
*Cag*A: Cytotoxin-associated gene A
CHBAH: Chris Hani Baragwanath Academic Hospital
DFG: Deutsche Forschungsgemeinschaft
DNA: Deoxyribonucleic acid
*Dup*A: Duodenal ulcer promoting gene A
GIT: Gastro intestinal tract
PCR: Polymerase chain reaction
*Vac*A (m and s): *Vac*A (middle and signal)
*Vac*A: Vacuolating cytotoxin gene A
