# Supplementary material for: Detection of Helicobacter pylori and its virulence genes (cagA, dupA, and vacA) among patients with gastroduodenal diseases in Chris Hani Baragwanath Academic Hospital, South Africa
Source: BMC Gastroenterol. 2019 May 14;19:73. doi: 10.1186/s12876-019-0986-0 (PMC6518451; doi:10.1186/s12876-019-0986-0)
Supplement: Supplementary file 1 — Patient questionnaire. Contains patients investigation form, gastroenterology result sheet and consent form. (PDF 420 kb) [file 12876_2019_986_MOESM1_ESM.pdf]

# PATIENT QUESTIONNAIRE



DFG
Africa Initiative

Deutsche
Forschungsgemeinschaft
DFG

## PATIENT QUESTIONNAIRE

ID SA - 2 - ___ ___ ___
Patient

Hospital: ID2 = Baragwanath Hospital, Johannesburg

**Coordinators**

Munich, Germany
Dr. Ute Breithaupt
Max von Pettenkofer-Institute
Mail: breithaupt@mvp.uni-muenchen.de

LMU

Alice, South Africa
Dr. Anna M. Clarke
University of Fort Hare
Mail: AClarke@ufh.ac.za

University of Fort Hare
Together in Excellence

Lagos, Nigeria
Dr. Stella I. Smith
Nigerian Institute of Medical Research (NIMR)
Mail: stellaismith@yahoo.com

Research for National Health

Treating gastroenterologist:

| | | | | | | | | | | | | | | | | | |
|---|---|---|---|---|---|---|---|---|---|---|---|---|---|---|---|---|---|
| | | | | | | | | | | | | | | | | | |

Gender

☐ Female ☐ Male

First Name

| | | | | | | | | | | | | | | | | | |
|---|---|---|---|---|---|---|---|---|---|---|---|---|---|---|---|---|---|

Middle Name

| | | | | | | | | | | | | | | | | | |
|---|---|---|---|---|---|---|---|---|---|---|---|---|---|---|---|---|---|

Last Name

| | | | | | | | | | | | | | | | | | |
|---|---|---|---|---|---|---|---|---|---|---|---|---|---|---|---|---|---|

Date of birth

☐☐ / ☐☐ / ☐☐☐☐

month day year

Nationality

| | | | | | | | | | | | | | | | | | |
|---|---|---|---|---|---|---|---|---|---|---|---|---|---|---|---|---|---|

If not born in South Africa: Date of moving to South Africa and from where:

☐☐ / ☐☐☐☐

month year

| | | | | | | | | | | | | | | | | | |
|---|---|---|---|---|---|---|---|---|---|---|---|---|---|---|---|---|---|

From where did you move to South Africa?

**1. Have you stayed in other countries for more than 1 month during the last 5 years?**

□ yes □ no □ don't know

**If yes,** please specify the countries

**2. Highest education** (Please select only one answer)

- □ No formal
- □ Koranic
- □ Primary
- □ Secondary
- □ Post secondary

**3. Habits**

- □ Smoking
- □ Occasionally alcohol
- □ Regularly alcohol

**4. Main occupation** (Please select only one answer)

- □ Working with sick people
- □ Working with children
- □ Working with animals

  **If yes,** please specify the species

- □ Others

**5. Do you have any occupational exposure to people with gastric disorders?**

□ yes □ no □ don't know

**6. Source of drinking water** (several answers are possible)

- ☐ Pipe-borne water
- ☐ Well water
- ☐ River
- ☐ Borehole
- ☐ Bottled water
- ☐ Others

Please specify "others"

| | | | | | | | | | | | | | | | |
|---|---|---|---|---|---|---|---|---|---|---|---|---|---|---|---|

**7. Do you have children?**

- ☐ 0
- ☐ 1
- ☐ 2
- ☐ 3
- ☐ More than 3

**If you have children**, do / did you give them pre-masticate food for them?

☐ yes ☐ no

**8. With how many people <u>did</u> you grow up (within one apartment/house)?**

- ☐ 0
- ☐ 1
- ☐ 2
- ☐ 3
- ☐ More than 3

**9. With how many people do you <u>currently</u> live together (within one apartment/house)?**

- ☐ 0
- ☐ 1
- ☐ 2
- ☐ 3
- ☐ More than 3

**10. Any history of ulcer / gastritis within the family?**

| Yes | No | Don't know | |
|---|---|---|---|
| ☐ | ☐ | ☐ | Father |
| ☐ | ☐ | ☐ | Mother |
| ☐ | ☐ | ☐ | Siblings |
| ☐ | ☐ | ☐ | Grandparents |
| ☐ | ☐ | ☐ | Children |

**11. Any history of gastric cancer within the family?**

| Yes | No | Don't know | |
|---|---|---|---|
| ☐ | ☐ | ☐ | Father |
| ☐ | ☐ | ☐ | Mother |
| ☐ | ☐ | ☐ | Siblings |
| ☐ | ☐ | ☐ | Grandparents |
| ☐ | ☐ | ☐ | Children |

# GASTROENTEROLOGY FORM



DFG
Africa Initiative

Deutsche
Forschungsgemeinschaft
DFG

## GASTROENTEROLOGY

ID SA - 2 - _ _ _
Patient

**Hospital**

ID2 = Baragwanath Hospital, Johannesburg

**Coordinators**

Munich, Germany Dr. Ute Breithaupt
Max von Pettenkofer-Institute
Mail: breithaupt@mvp.uni-muenchen.de

Lagos, South Africa Dr. Anna M. Clarke
University of Fort Hare
Mail: AClarke@ufh.ac.za

University of Fort Hare
Together in Excellence

Gender

☐ Female ☐ Male

First Name

| | | | | | | | | | | | | | | | | | |
|---|---|---|---|---|---|---|---|---|---|---|---|---|---|---|---|---|---|
| | | | | | | | | | | | | | | | | | |

Middle Name

| | | | | | | | | | | | | | | | | | |
|---|---|---|---|---|---|---|---|---|---|---|---|---|---|---|---|---|---|
| | | | | | | | | | | | | | | | | | |

Last Name

| | | | | | | | | | | | | | | | | | |
|---|---|---|---|---|---|---|---|---|---|---|---|---|---|---|---|---|---|
| | | | | | | | | | | | | | | | | | |

Date of performing the gastroscopy:

☐☐ / ☐☐ / ☐☐☐☐

month day year

**Did anyone of your relatives take part in this study?**

☐ yes ☐ no

**If yes:**

First Name

| | | | | | | | | | | | | | | | |
|---|---|---|---|---|---|---|---|---|---|---|---|---|---|---|---|
| | | | | | | | | | | | | | | | |

Last Name

| | | | | | | | | | | | | | | | |
|---|---|---|---|---|---|---|---|---|---|---|---|---|---|---|---|
| | | | | | | | | | | | | | | | |

Relatedness

| | | | | | | | | | | | | | | | |
|---|---|---|---|---|---|---|---|---|---|---|---|---|---|---|---|
| | | | | | | | | | | | | | | | |

In which hospital was this relative treated?

| | | | | | | | | | | | | | | | |
|---|---|---|---|---|---|---|---|---|---|---|---|---|---|---|---|
| | | | | | | | | | | | | | | | |

1. a. PCR result positive ☐ negative ☐

   b. Culture result positive ☐ negative ☐

2. Number of taken biopsies

| Antrum | Corpus |
|---|---|
| ☐ 0 | ☐ 0 |
| ☐ 1 | ☐ 1 |
| ☐ 2 | ☐ 2 |
| ☐ 3 | ☐ 3 |

3. Endoscopic assessment in <u>stomach</u>

| Yes | No | |
|---|---|---|
| ☐ | ☐ | Normal mucosa |
| ☐ | ☐ | Erosion |
| ☐ | ☐ | Aphtous ulcers |
| ☐ | ☐ | Ulcers |
| ☐ | ☐ | Cancer |
| ☐ | ☐ | Mucosal atrophy |
| ☐ | ☐ | Excrescence |
| ☐ | ☐ | Polyps smaller than 1cm |
| ☐ | ☐ | Polyps bigger than 1cm |
| ☐ | ☐ | Haemorrhages |
| ☐ | ☐ | Hyperaemic |
| ☐ | ☐ | Hypoaemic |
| ☐ | ☐ | Oedematous |
| ☐ | ☐ | Others |

Please specify “others”

| | | | | | | | | | | | | | | | |
|---|---|---|---|---|---|---|---|---|---|---|---|---|---|---|---|
| | | | | | | | | | | | | | | | |

4. Endoscopic assessment in <u>oesophagogastric junction</u>

| Yes | No | |
|---|---|---|
| ☐ | ☐ | Normal |
| ☐ | ☐ | Reddening |
| ☐ | ☐ | Irregular Z-line |
| ☐ | ☐ | Salmon-pink tongue like |
| ☐ | ☐ | segment |
| ☐ | ☐ | Others |

Please specify “others”

| | | | | | | | | | | | | | | | |
|---|---|---|---|---|---|---|---|---|---|---|---|---|---|---|---|
| | | | | | | | | | | | | | | | |

| ID | SA | - |  | - |  |  |  |
|---|---|---|---|---|---|---|---|
|  |  |  | ID |  | patient |  |  |

# Research Subject Information and Consent form

**Title: Analysis of *Helicobacter pylori* infections in Africa, bacterial virulence factors but lack of pathology**

| | |
|---|---|
| Sponsor: | DFG |
| Investigator: | Dr. Anna M. Clarke |
| ID1: | Livingston Hospital, Port Elizabeth |
| ID2: | Baragwanath Hospital, Johannesburg |
| Co-investigators: | Prof. Rainer Haas, Dr. Ute Breithaupt |

You are invited to participate in a research study. You may decide whether or not you want to participate. Please take time to make your decision. Carefully read the following and ask questions you may have. You may take home an unsigned copy of this consent form to think or discuss with your family and friends before making your decision.

The purpose of this study is to find out the best method that we can use to characterize a germ that causes ulcer and later stomach cancer. The study is expected to last for three years and a total of 500 patients would be enrolled in the study.

The study will involve collecting biopsies from 500 patients already billed by the gastroenterologist for endoscope for the culture of this germ. The germ will be detected and necessary treatment would be conveyed to your physician who would prescribe it to you. In addition, various DNA tests would be done on the samples to enable prompt diagnosis and treatment of the germ.

The biopsy procedure has its discomfort that is why we would not collect samples from you for the research. We need your consent to collect the biopsies when you must have visited the gastroenterologist and he recommends endoscopy.

The tests would be done free of cost, except of course for the endoscopy where you must have visited your physician to carry out the endoscopy.

Taking part in this research is voluntary and will benefit you because the tests would be done free of costs and the ailment troubling you would be diagnosed faster and the correct treatment would be carried out with faster cure and healing expected to take place.

Confidentiality

The data and results relevant to the study will be kept out of the reach of people other than the investigators and sponsors of the research. The samples and the results would be used for research purposes only.

Whom to contact

In case of any further information, questions or clarification please contact:

1) Dr. Anna M. Clarke
University of Hare, Alice Campus
5700 Alice, SOUTH AFRICA
Mail: AClarke@ufh.ac.za
Tel: +27 466225468

2) Dr. Ute Breithaupt
Max von Pettenkofer-Institute
80336 Munich, GERMANY
Mail: breithaupt@mvp.uni-muenchen.de
Tel: +49 89 2180 72936

Your participation is completely voluntary and you have a right to refuse to be in this study. You can withdraw your participation at any time after giving your consent. Such decision will not affect your current or future medical care in any way.

If you are willing to volunteer for this research please sign or thumbprint below

| | | |
|---|---|---|
| ____________________ | ____________ | ____________ |
| Patient's name | Signature | Date |
| ____________________ | ____________ | ____________ |
| Witness (if necessary) | Signature | Date |
