# Supplementary material for: Detection of Helicobacter pylori and its virulence genes (cagA, dupA, and vacA) among patients with gastroduodenal diseases in Chris Hani Baragwanath Academic Hospital, South Africa
Source: BMC Gastroenterol. 2019 May 14;19:73. doi: 10.1186/s12876-019-0986-0 (PMC6518451; doi:10.1186/s12876-019-0986-0)
Supplement: Supplementary file 2 — Ethics certificate. Contains ethics certificates from the CHBAH and University of Fort Hare. (PDF 154 kb) [file 12876_2019_986_MOESM2_ESM.pdf]

University of Fort Hare
Together in Excellence

ETHICAL CLEARANCE CERTIFICATE
REC-270710-028-RA Level 01

Certificate Reference Number: ANA01 (Project)

Project title: **Analysis of virulence factors of South Africa and Nigeria H. Pylori isolates and comparison to patient pathology**

Nature of Project: Independent project

Principal Researcher: Dr A Clarke
Sub researchers Prof R Haas
Prof Stella I Smith

Supervisor: N/A

Co-supervisor: N/A

On behalf of the University of Fort Hare's Research Ethics Committee (UREC) I hereby give ethical approval in respect of the undertakings contained in the above-mentioned project and research instrument(s). Should any other instruments be used, these require separate authorization. The Researcher may therefore commence with the research as from the date of this certificate, using the reference number indicated above.

Please note that the UREC must be informed immediately of

- Any material change in the conditions or undertakings mentioned in the document

- Any material breaches of ethical undertakings or events that impact upon the ethical conduct of the research

The Principal Researcher must report to the UREC in the prescribed format, where applicable, annually, and at the end of the project, in respect of ethical compliance.

Special conditions: Research that includes children as per the official regulations of the act must take the following into account:

Note: The UREC is aware of the provisions of s71 of the National Health Act 61 of 2003 and that matters pertaining to obtaining the Minister's consent are under discussion and remain unresolved. Nonetheless, as was decided at a meeting between the National Health Research Ethics Committee and stakeholders on 6 June 2013, university ethics committees may continue to grant ethical clearance for research involving children without the Minister's consent, provided that the prescripts of the previous rules have been met. This certificate is granted in terms of this agreement.

The UREC retains the right to

- Withdraw or amend this Ethical Clearance Certificate if
  - Any unethical principal or practices are revealed or suspected
  - Relevant information has been withheld or misrepresented
  - Regulatory changes of whatsoever nature so require
  - The conditions contained in the Certificate have not been adhered to

- Request access to any information or data at any time during the course or after completion of the project.

- In addition to the need to comply with the highest level of ethical conduct principle investigators must report back annually as an evaluation and monitoring mechanism on the progress being made by the research. Such a report must be sent to the Dean of Research's office

The Ethics Committee wished you well in your research.

Yours sincerely

Professor Gideon de Wet
Dean of Research

12 February 2015

R14/49 Prof Reidwan Ally and Prof Anna Clarke

HUMAN RESEARCH ETHICS COMMITTEE (MEDICAL)

CLEARANCE CERTIFICATE NO. M160228

NAME: (Principal Investigator) Prof Reidwan Ally and Prof Anna Clarke

DEPARTMENT: Internal Medicine
Chris Hani Baragwanath Academic Hospital
University of the Witwatersrand
University of Fort hare

PROJECT TITLE: Analysis of Virulence Factors of South African and Nigerian Helibacter Pylori Isolates and Comparison to Patient Pathology

DATE CONSIDERED: 26/02/2016

DECISION: Approved unconditionally

CONDITIONS:

SUPERVISOR:

APPROVED BY: Professor P. Cleaton-Jones, Chairperson, HREC (Medical)

DATE OF APPROVAL: 04/04/2016

This clearance certificate is valid for 5 years from date of approval. Extension may be applied for.

DECLARATION OF INVESTIGATORS

To be completed in duplicate and **ONE COPY** returned to the Research Office Secretary in Room 10004, 10th floor, Senate House/2nd Floor, Phillip Tobias Building, Parktown, University of the Witwatersrand. I/we fully understand the conditions under which I am/we are authorized to carry out the above-mentioned research and I/we undertake to ensure compliance with these conditions. Should any departure be contemplated, from the research protocol as approved, I/we undertake to resubmit the application to the Committee. **I agree to submit a yearly progress report**.

Principal Investigator Signature ____________________ Date ____________________

PLEASE QUOTE THE PROTOCOL NUMBER IN ALL ENQUIRIES
